# Cloning and Characterization of the Gene Encoding *HMGS* Synthase in *Polygonatum sibiricum*

**DOI:** 10.1155/2022/7441296

**Published:** 2022-10-07

**Authors:** Yujie Jiang, Dekai Wang, Kangjing Wu, Feifeng Wang, Qingwen Yang, Ruilian Han, Zongsuo Liang, Qiaojun Jia

**Affiliations:** ^1^College of Life Sciences and Medicine, Zhejiang Sci-Tech University, Hangzhou Zhejiang 31018, China; ^2^Key Laboratory of Plant Secondary Metabolism and Regulation of Zhejiang Province, Hangzhou Zhejiang 31018, China

## Abstract

The saponins of *Polygonatum sibiricum* had many pharmacological activities such as antitumor, antioxidation, and blood sugar lowering, which were synthesized by two pathways: mevalonate (MVA) and methylerythritol phosphate (MEP). 3-Hydroxy-3-methylglutaryl coenzyme A synthase (HMGS) was the key enzyme in the MVA synthesis pathway, and its expression level may affect the accumulation of saponins which were the main active ingredients of *P. sibiricum*. In this study, we successfully cloned *HMGS1* and *HMGS2* from *P. sibiricum* and their sequence similarity was 93.71% with 89 different sites. The multiple sequence alignment results indicated that the N-terminal sequences of HMGS were conserved. Phylogenetic analysis showed that *P. sibiricum*, *A. officinalis*, *N. tazetta*, *D. nobile*, and other relatives had a common evolutionary ancestor. The expression levels of both *HMGSs* and the total saponin content in different tissues revealed that *HMGS* expression in rhizomes was positively correlated with total saponin content. Further study of the abiotic stress effect of Methyl Jasmonate (MeJA) demonstrated that the expression of *HMGS1* and *HMGS2* genes was induced by MeJA, peaked at 24 h, and fell by 48 h. Our present findings would provide a blueprint for future studies of *HMGS* and its role in triterpenoid biosynthesis in *P. sibiricum*.

## 1. Introduction

**Figure 1:**
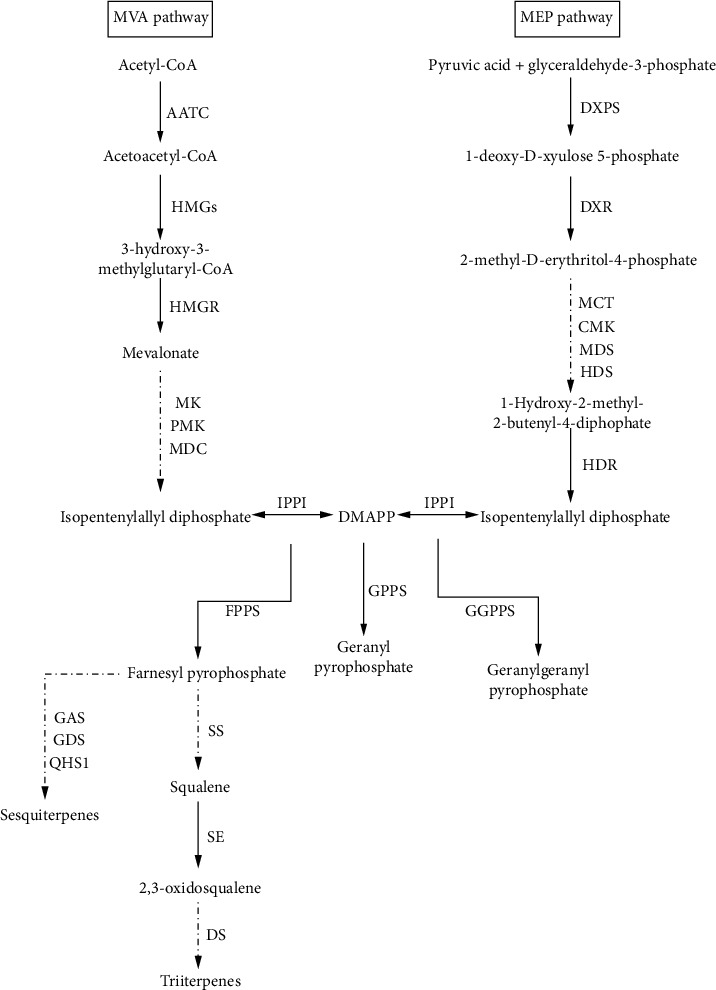
Synthetic pathway of plant terpenes. AACT: acetoacetyl-CoA acyltransferase; HMGS: 3-hydroxy-3-methylglutaryl-CoA synthase; HMGR: 3-hydroxy-3-methylglutaryl-CoA reductase; MK: mevalonic acid kinase; PMK: phosphomevalonate kinase; MDC: mevalonate-5-pyrophosphate decarboxylase; DXPS: 1-deoxy-D-xylulose-5-phosphate synthase; DXR: 1-deoxy-D-xylulose-5-phosphate reductoisomerase; MCT: 2-C-methyl-d-erythritol 4-phosphate cytidylyltransferase; CMK: 4-(cytidine 5′-diphospho)-2-C-methyl-D-erythritol kinase; MDS: 2-C-methyl-D-erythritol 2,4-cyclodiphosphate synthase; HDS: 4-hydroxy-3-methylbut-2-enyldiphosphate synthase; IPPI: IPP isomerase; GPPS: geranyl pyrophosphate synthase; FPPS: farnesyl pyrophosphate synthase; GGPPS: geranylgeranyl diphosphate synthase; SS: squalene synthase; SE: squalene epoxidase; DS: dammarenediol-II synthase; HDR: 4-hydroxy-3-methylbut-2-enyldiphosphate reductase; GAS: germacrene A synthase; GDS: germacrene D synthase; QHS1: beta-caryophyllene synthase.

*Polygonatum sibiricum* is a perennial herb in Liliaceae, mainly distributed in north temperate countries such as China and North Korea. *Polygonatum sibiricum* is a great reservoir of various secondary metabolites including steroid saponins, triterpenoid saponins, polysaccharides, flavonoids, and volatile oils, among which steroidal saponins are the main ones. Due to the variety of saponin skeleton types, *P. sibiricum* has extremely high medicinal value, such as lowering blood sugar and lipids, improving immunity, and antitumor effects [1–4]. The biosynthesis of saponins can be divided into three stages: terpenoid backbone biosynthesis, sesquiterpenoid and triterpenoid biosynthesis, and steroid biosynthesis. Terpenoids of plants are synthesized by two independent pathways (Figure 1): the mevalonic acid pathway (MVA) and the 2-C-methyl-D-eryth -ritol-4-phosphate pathway (MEP). The two pathways are used to synthesize saponin precursor compounds, isopentenyl pyrophosphate (IPP), and dimethylallyl pyrophosphate (DMAPP). IPP and DMAPP, under the action of terpenoid synthetases, generate a series of intermediate compounds: geranyl pyrophosphate (GPP), farnesyl pyrophosphate (FPP), geranylgeranyl pyrophosphate (GGPP), etc., and finally generate monoterpenes, sesquiterpenes, and diterpenes. In the MVA pathway, HMGS and HMGR are the key enzymes involved in the irreversible catalytic reactions of terpenoid biosynthesis in plants [5, 6].

The content of chemical components in medicinal plants may be related to biosynthetic genes. The HMGS is located in the cytoplasm of plants [7] and is closely related to seed germination, early development, pollen fertility, and phytosterol biosynthesis [8, 9]. Through overexpression of the *HMGS* gene, the sterol contents of *Arabidopsis thaliana* and *Nicotiana tabacum* are increased [10, 11]. In addition, overexpression of *HMGS* upregulates the expression levels of five genes (*HMGR*, *SMT2*, *DWF1*, *CYP710A1*, and *BR6OX2*) in the MVA pathway of isoprenoid biosynthesis, leading to enhanced sterol content and stress tolerance in *Arabidopsis* [11]. Up to now, the *HMGS* genes in many plants have been successfully isolated and cloned, such as *Taxus chinensis* [12], *Hedera nepalensis* [13], and *Antirrhinum majus* [14]. However, most of the genes including *HMGS* involved in the terpenoid biosynthetic pathway are not identified in *P. sibiricum*.

In order to explore the mechanism of terpenoid biosynthetic genes and saponin content, we attempted to isolate the full-length cDNA sequence of the *HMGS* gene from transcriptome data of *P. sibiricum*. We also presented the characterization, evolution, and transcription profiling of HMGSs, as an initial step to investigate the functional role in *P. sibiricum* in the future. In addition, the expression level of HMGSs in aseptic seedlings induced by Methyl Jasmonate (MeJA) was investigated.

## 2. Materials and Methods

### 2.1. Material

The four-year-old *P. sibiricum* Red. was harvested in Polygonatum Planting Base of Buchang Pharmaceutical Group (Lueyang County, Shaanxi Province) in September 2019. Because the rhizome of *P. sibiricum* Red. grows one section per year, the rhizome born in 2019 was called the first section of rhizome, while the one born in 2018 was called the second section of rhizome and so on. Different sections of rhizome, fruit, leaves, and stems were collected to extract RNA and detect saponin content. The tissue culture seedlings were obtained from the seeds of *P. sibiricum* Red. as described by Zhang et al. [4] .The *seedlings* were cultured and rooted for 30 days. Then, they were treated with Methyl Jasmonate (MeJA) at a concentration of 400 *μ*m·L^−1^. The aerial parts of the seedling were harvested for RNA isolation at 0, 12, 24, and 48 h posttreatment, respectively, and stored at -80°C for further analyses.

### 2.2. Method

#### 2.2.1. RNA Isolation and cDNA Chain Synthesis

The total RNA from the aerial parts of seedlings with MeJA treatment and the fruit, leaves, stems, and different sections of rhizomes of the four-year-old *P. sibiricum in* Red. were extracted using the RNA Isolation Kit (Tiangen, Beijing, China) according to the manufacturer's instructions. The first-strand cDNA was synthesized by the Reverse Transcription Kit (Vazyme, Nanjing, China) and was stored at -20°C until use.

#### 2.2.2. Cloning of the *HMGS* Gene

The primers of *HMGS* were designed according to the gene annotation of *P. sibiricum* in the transcriptome database (Table 1). The PCR reaction was as follows: initial denaturation at 94°C for 2 min, 35 cycles of denaturation at 94°C for 1 min, annealing at 55°C for 1 min, extension at 72°C for 1.5 min, final extension at 72°C for 5 min, and rapid cooling at 10°C. The amplification products were run in 1% agarose gels, and the target fragments were purified by TIANgel MiDi Purification Kit (Tiangen, Beijing, China). Then, the purified fragments were cloned into the pMD19-T vector. The vector was transformed into *E. coli* DH5*α* competent cells (Weidi, Shanghai, China) by heat shock. The positive clone was selected in Luria Bertani (LB) medium with ampicillin (100 mg/L) at 37°C in the dark and was confirmed using sequencing by Zhejiang Youkang Biotechnology Co., Ltd.

#### 2.2.3. Bioinformatics Analysis and Molecular Evolution Analysis

The molecular weight, structural stability, theoretical isoelectric point, and amino acid composition of *HMGS1* and *HMGS2* were analyzed by the online software ProtParam (http://web.expasy.org/protparam/). SOPMA (https://npsa-prabi.ibcp.fr/cgi- bin/npsa_automat.pl?page=npsa_sopma.html) and SWISS-MODEL (http://swissm-odel.expasy.org/) were used to predict the secondary structure and three-dimensional model of HMGS. The differences between *HMGS1* and HMGS2 in nucleic acid sequences and protein sequences were analyzed by DNAMAN software. The multiple sequences and phylogenetic analysis of protein sequences were analyzed by Clustal W and Clustal X, and the protein sequences with high similarity (>80%) to other species were obtained in the NCBI online database.

#### 2.2.4. Quantitative Real-Time PCR

The qRT-PCR was performed using a ChamQ Green I SYBR qPCR Master Mix (Q711) qPCR Kit (Vazyme, Nanjing, China) with the QuantStudio™ 6 Flex Real-Time PCR System (Applied Biosystems, Thermo, Singapore) under the following conditions [15]: 95°C for 30 s, 40 cycles of 95°C for 5 s, and 55°C for 30 s, followed by 95°C for 15 s, 60°C for 1 min, and 95°C for 15 s to obtain melt curves. The real-time PCR assays were performed in triplicate for each cDNA sample. The expression of 18s rRNA for *P. sibiricum* Red. was employed as the reference gene. To determine the transcriptions levels of *HMGS1* and *HMGS2*, the expression level was calculated using the 2^-*ΔΔ*Ct^ method [16].  Table 2 lists the oligonucleotide sequences used for quantitative RT-PCR.

#### 2.2.5. Extraction of Total Saponins and Determination of the Content

The total saponins from fruits, leaves, stems, and different sections of rhizomes were extracted by ultrasonic extraction under the conditions of 70% ethanol concentration, 1 : 15 solid-liquid ratio, 500 W ultrasonic power, 30 min extraction time, and 3 extraction times. The total saponin contents were determined as described by Yu et al. [17].

## 3. Results

### 3.1. Cloning and Sequence Analysis of *HMGS*

**Figure 2:**
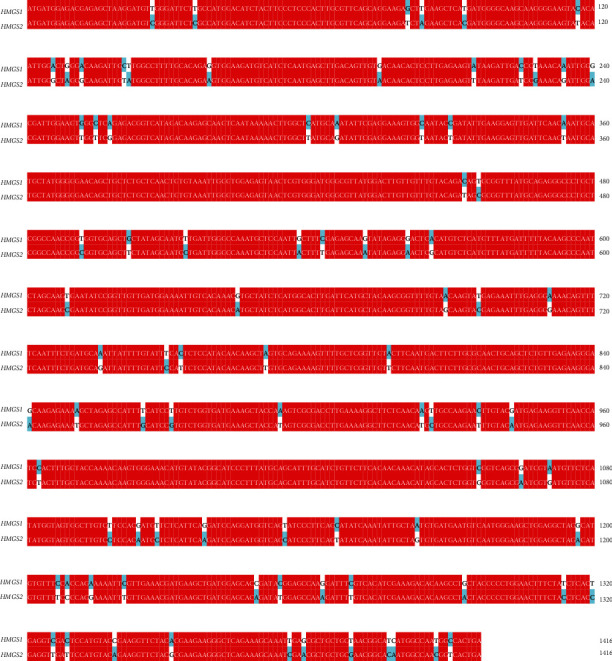
The differences of the nucleic acid sequence between *HMGS1* and *HMGS2*.

**Figure 3:**
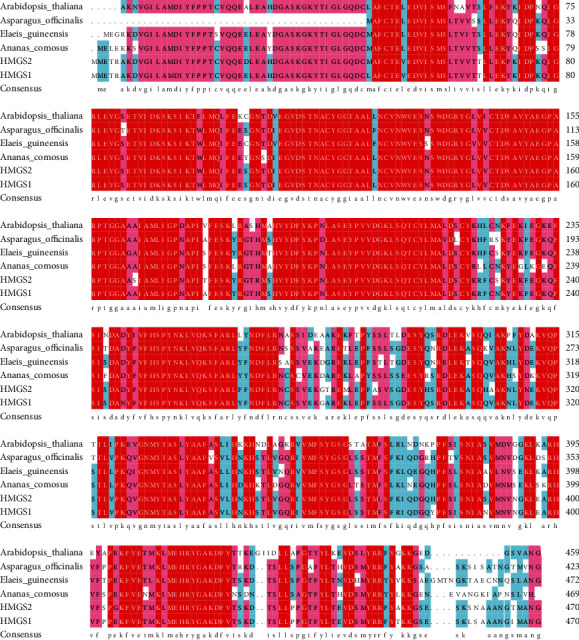
The multiple alignment of *P. sibiricum* Red. HMGS amino acid sequence with other and HMGS proteins.

The PCR products of *HMGS1* and *HMGS2* were sequenced, and the results showed that the cDNA sequences of the PCR products were 1470 bp and 1416 bp, respectively (supplement (available here)). The similarity of the nucleic acid sequence between *HMGS1* and *HMGS2* was 93.71% with 89 different sites (Figure 2). In addition, both of them contained a 1416 bp open reading frame encoding a polypeptide of 471 amino acids with an isoelectric point (pI) of 5.02. The similarity of protein sequence between them was 94.27% with 27 different sites (Figure 3). The calculated molecular weight of HMGS1 and HMGS2 was about 116004.98 KDa and 116102.99 KDa, and stability coefficients were 40.88 and 41.01, respectively.

The multiple sequence alignment of protein sequence showed that the HMGS protein sequences of *P. sibiricum* Red. had high homology with other HMGSs such as *Arabidopsis thaliana* (79.27%; 76.50%), *Asparagus officinalis* (88.30%; 84.89%), *Elaeis guineensis* (85.44%; 83.54%), and *Ananas comosus* (84.04%; 82.55%) (Figure 3). Their amino acid sequences in N-terminalis were highly conserved.

### 3.2. Analysis of Secondary and Tertiary Structure

**Figure 4:**
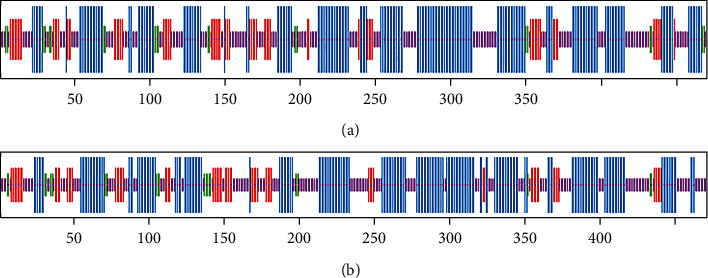
Prediction of the secondary structure of the protein encoded by the *HMGS* gene. (a) *HMGS1*; (b) *HMGS2*. The blue line represents *α*-helix; the green line represents *β*-turn; the purple line represents random coil; and the red line represents extended strand.

The secondary structure of HMGS1 and HMGS2 was analyzed by SOPMA which predicted the *α*-helix, extended strand, *β*-turn, and random coil (Figure 4). In the designed secondary structure of HMGS proteins, the putative HMGS1 peptide contained 47.02% of *α*-helix, 13.62% of the extended strand, 4.47% of *β*-turn, and 34.89% of a random coil. The HMGS2 structure possessed 44.37% of *α*-helix, 14.44% of the extended strand, 4.46% of *β*-turn, and 36.73% of a random coil. Both of the HMGS proteins revealed that the *α*-helix and random coil constituted interlaced domination of the main part of the secondary structure.

**Figure 5:**
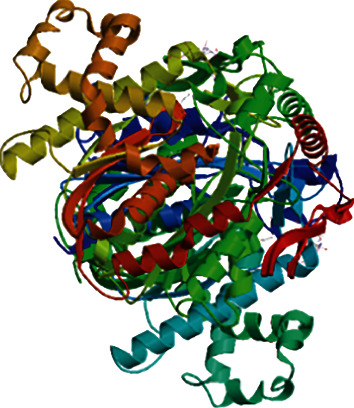
The tertiary structure of the protein encoded by the *HMGS* gene.

**Figure 6:**
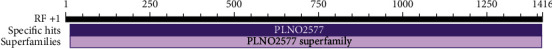
The conserved domain structure of the protein encoded by the *HMGS* gene.

The three-dimensional models of HMGS1 and HMGS2 were analyzed by SWISS-MODEL, and the results showed that the sequence identity of HMGS1 and HMGS2 to the template sequence (*Brassica juncea*) was 82.81% and 80.36%, respectively, and the coverage was 0.95 (Figure 5). The sequence was described as 3-hydroxy-3methyl-glutaryl- CoA synthase (HMG-CoA), which was consistent with the cloned gene. The HMGS catalytic domain can form a homodimer with two acetyl-CoA ligands. Through further prediction of the conserved domain structure of HMGS1 and HMGS2, the proteins had a landmark domain matching with other model organisms and were HMG-CoA synthase, which belonged to the PLN02577 superfamily (Figure 6).

### 3.3. Molecular Evolution Analysis

**Figure 7:**
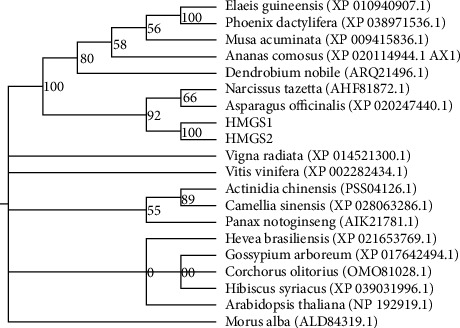
Phylogenetic tree analysis of protein encoded by *HMGS* genes.

A phylogenetic tree was constructed based on the deduced amino acid sequences of both HMGSs and its homologous species to investigate their evolutionary relationships (Figure 7). The results showed that HMGS1 and HMGS2 of *P. sibiricum* Red. were clustered with all the monocotyledonous plants and were farther away from the dicotyledonous plants. Furthermore, both HMGSs formed a branch of its own and were closely related to *Asparagus officinalis* (XP020247440.1) and *Narcissus tazetta* (AHF81872.1) than other species. Further phylogenetic analysis of HMGS with the above-mentioned HMGSs showed that the *P. sibiricum* Red., *A. officinalis*, *N. tazetta*, *Dendrobium nobile*, *Ananas comosus*, *Musa acuminata*, *Elaeis guineensis*, and *Phoenix dactylifera* have a common evolutionary ancestor.

### 3.4. Expression of *HMGS* in Tissues

**Figure 8:**
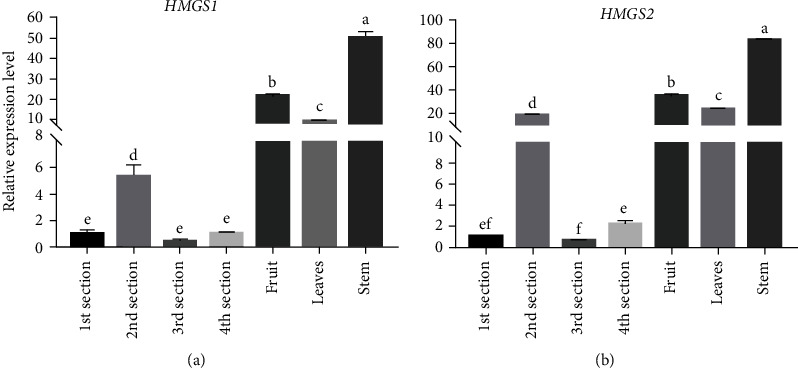
Relative expression of *HMGS1* and *HMGS2* in different tissues. Values are mean ± SD of three biological replicates. Samples with different letters are significantly different (*P* < 0.01; Tukey's test).

As shown in Figure 8, both *HMGS1* and *HMGS2* were expressed constitutively in all tissues examined with different levels. Their expression patterns in different tissues were consistent, which were highly expressed in aerial parts. In addition, the highest transcript levels of both genes were observed in the stem, followed by fruit. Particularly, the expression levels of both genes in the second rhizome were higher than those of the other three ones.

### 3.5. Total Saponin Content and Its Correlation with the Expression of *HMGSs*

**Figure 9:**
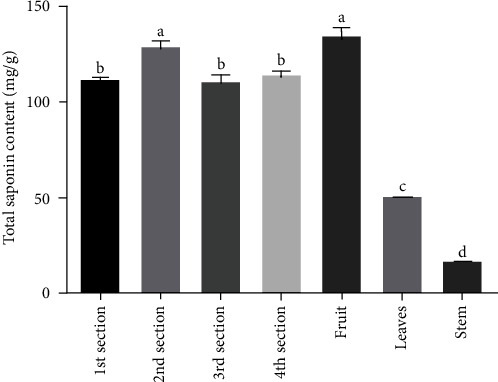
Total saponin content of *P.* sibiricum in in different ages. Values are mean ± SD of three biological replicates. Samples with different letters are significantly different (*P* < 0.01; Tukey's test).

Total saponin content analysis showed that the rhizome and fruit were rich in saponin, while the saponin content in the stem was the lowest (Figure 9). What is more, the total saponin content in the second section of rhizomes was the highest, and there was no significant difference in the other three rhizomes.

There was no correlation between *HMGS* gene expression and total saponin content in aerial parts; but in rhizomes, *HMGS* gene expression was significantly correlated with total saponin content (Table 3).

### 3.6. Expression Profiles of *HMGS* under Induction of MeJA

**Figure 10:**
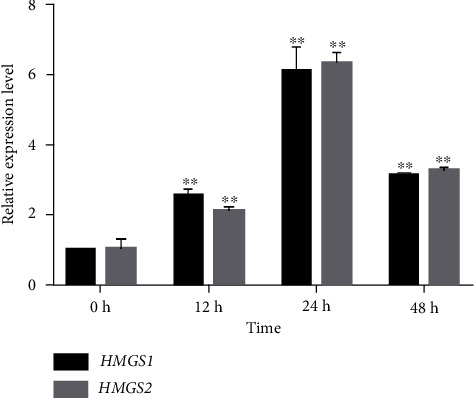
Temporal and spatial expression of *HMGSs* in Polygonatum seedlings. Note: different treatment times are compared with control: *P* < 0.05 is represented by ∗; *P* < 0.01 is represented by ∗∗.

To understand the expression pattern of *HMGS* under the signal molecule MeJA in *P. sibiricum* Red., the expression levels of *HMGSs* were measured by qRT-PCR analysis. As showed in Figure 10, both *HMGS1* and *HMGS2* were obviously induced by MeJA, and their expression levels were similar. *HMGS* expressions were significantly increased 12 h posttreatment, peaked 24 h posttreatment, and decreased at 48 h after treatment but still maintained at an increased level at 48 h compared with the control.

## 4. Discussion

HMGS is a rate-limiting enzyme that catalyzes an important step in MVA biosynthesis [18] and plays a key role in the synthesis of plant terpenoids, which is essential not only for plant growth and development but also for plant adaptation to harsh environmental conditions. Afroz et al. cloned a *HMGS* gene from *Centella asiatica*, and the complementation test showed that the *CaHMGS* gene encodes functional protein that catalyzed the synthesis of mevalonate in the MVA pathway [19]. The overexpression of the *HMGS* gene resulted in the sterol accumulation in *Arabidopsis* due to its upregulation of *HMGR*, *SMT2*, *DWF1*, *CYP710A1*, and *BR6OX2* in MVA-dependent steroid biosynthesis [11]. Since saponin was the main component of *P. sibiricum*, we cloned the *HMGS* gene.

In this work, we successfully isolated the cDNA of *HMGS1* and *HMGS2* genes from *P. sibiricum* Red., which was 1470 bp and 1416 bp, respectively. Li et al. [20] found that the number of unigene HMGS in *Polygonatum cyrtonema Hua* was 1. SOPMA predicted the secondary structure of HMGS, which was mainly *α*-helix and random coils, reaching 81.91% and 81.10%, respectively (Figure 4). The amino acid sequence upon BLAST showed high similarities with HMGSs of other plants, such as *A. thaliana*, *A. officinalis*, *E. guineensis*, and *A. comosus* (Figure 3), which further verified that *P. sibiricum* HMGSs belonged to the plant HMGS family. Furthermore, the phylogenetic analysis showed that *HMGS1* and *HMGS2* of *P. sibiricum* Red. (monocotyledonous plant) were related with the HMGSs from all the monocotyledonous plants (Figure 7). Cheng et al. found that *Chamaemelum nobile* (dicotyledon plant) *HMGS* clustered with the HMGS of Asteraceae in the dicotyledon clade. The result of phylogenetic analysis implied that *P. sibiricum* Red., *A. officinalis*, *N. tazetta*, *D. nobile*, *A. comosus*, *M. acuminata*, *E. guineensis*, and *P. dactylifera* have a common evolutionary ancestor based on amino acid homology and their conserved domains [12].

And recently, the *HMGS* has been successfully cloned from a variety of plants and the spatial expression of *HMGS* varied in different tissues across different species. In *Taxus chinensis*, the expression level of *HMGS* in leaves and stems was higher than that in roots [12]. The expression profiles of *Tripterygium wilfordii HMGS* in the stems were the highest, followed by leaves, and the roots were the lowest [21]. By contrast, the expression levels of *HMGS* in *Chamaemelum nobile* were significantly higher in flowers and roots than those in stems and leaves [22]. Such spatial expression profile of *HMGS* might be associated with the content of sesquiterpenoids in different tissues of *Chamaemelum nobile* [22]. In our study, qRT-PCR was employed to investigate the expression profiles of the *HMGS* gene in different tissues, which revealed that both genes were constitutively expressed in all examined tissues (rhizomes, fruit, leaves, and stem) of *P. sibiricum*; such result was consistent with that in *Santalum album* and *Salvia miltiorrhiza* [23, 24]. Sun et al. [25] found that *MVD*, *IPPI*, and *FPPS* genes were significantly negatively correlated with the content of total saponins in the xylem of *Platycodon grandiflorum*, but in the root, those genes were significantly positively correlated with the content of platycodon saponin D. In the present study, the expression level of *HMGS* was the highest in the stems and was the lowest in the third rhizomes. However, the total saponin content was the highest in fruits and the lowest in stems, indicating that there was no synergy between the saponin content and expression levels of *HMGSs* in aerial parts. But in rhizomes, the gene expression level was synergistic with the total saponin content (Table 3). The following reasons were inferred: (1) the saponins were synthesized in different tissues and might be transported to fruits and rhizomes for storage [26]. (2) Sterols played an important role in maintaining cell morphology and maintaining membrane integrity. The high expression of the *HMGS* gene in the stem might be related to the self-protection of the cell to maintain the transport function of the stem [27].

MeJA, an important signal transduction molecule, regulated the synthesis of plant secondary metabolites through activating or inhibiting the activity of corresponding transcription factors, regulating the expression of key genes related to secondary metabolism, and affecting the activity of the enzymes. [28, 29]. The expression of *HMGS* was induced by MeJA in T*axus × media*, *Ganoderma lucidum*, *Aquilaria sinensis*, *P. tenuifolia*, *C*. *nobile*, and *Phellinus igniarius* [12, 22, 27, 30–32]. In the present study, the relative expression levels of *HMGS1* and *HMGS2* genes were induced at 12 h, 24 h, and 48 h after MeJA treatment (Figure 10). *HMGSs* expression was peaked at 24 h and fell by 48 h, which was consistent with previous reports in *Tripterygium wilfordii* [21], *Ganoderma lucidum* [32], *Arabidopsis* [11], *P. notoginseng* [33], Aquilaria sinensis (Lour.) Gilg [30], Roman chamomile [22], *Polygala tenuifolia* [31], and *Antirrhinum* [14]. Zhu et al. reported that the total saponin content in the MeJA-treated seedlings of the *P. notoginseng* was significantly higher than that in the control group. And Zhang et al. [34] concluded that MeJA could promote the production of plant secondary metabolites. Considering HMGS as one of the key enzymes involved in the saponin pathway in plant, the expression profile of *HMGSs* suggested that MeJA treatments might be an effective approach to promote the higher production of saponin in *P. sibiricum* Red..

## 5. Conclusions

In this study, *HMGS1* and *HMGS2* genes were successfully cloned from *P. sibiricum Red.*. Both putative HMGS proteins were analyzed in physical and chemical properties. The phylogenetic analysis indicated both HMGS1 and HMGS2 were closely related with other monocotyledonous plants. Association analysis showed that there was a positive correlation between the expression level of *HMGS* and total saponin content in the rhizome. Signal molecule MeJA upregulated the expression of *HMGSs*, indicating that such genes might participate in the response of signaling molecules to environmental stimuli in *P. sibiricum*. Therefore, *HMGS1* and *HMGS2* might be good candidate genes for the engineering of the saponin biosynthetic pathway. The successful cloning of *HMGS* genes could provide a theoretical basis for further research on its function and the accumulation of saponins in *P. sibiricum* Red.

## Figures and Tables

**Table 1:** Primers for the amplification of *HMGS*.

Primer name	Primer sequences (5′→3′)
HMGS1-F	CGTTAATCGAAGGAGGAGAGA
HMGS1-R	CAAAACCCATGCCACTGG
HMGS2-F	ATGATGGAGACGAGAGCTAAGGATG
HMGS2-R	TCAGTGACCGTTGGCCATTG

**Table 2:** Primers for real-time quantitative PCR.

Primers	Sequence (5′→3′)
HMGS1-Q-F	TTGGACTGGGACAAGATTGC
HMGS1-Q-R	TCGGTATTGCCACTTTCCTC
HMGS2-Q-F	GTGGGTCAGCGAATCGTAAT
HMGS2-Q-R	CCATATCTGTGCTCCATCAGC
18srRNA-F	CGAGTCTATAGCCTTGGCCG
18srRNA-R	ATCCGAACACTTCACCGGAC

**Table 3:** Correlation analysis based on total saponin content and gene expression in aerial parts and rhizomes.

	Aerial parts		Rhizomes	
	*HMGS1*	*HMGS2*	*HMGS1*	*HMGS2*
Total saponins content	-0.531	-0.529	0.975^∗^	0.981^∗^

∗ represents significant correlation in the *P* < 0.05 level.

## Data Availability

The data that support this study are available in the article.

## Supplementary Materials

Supplementary Materials Supplement: the sequences of the *HMGS1* and *HMGS2.*
