# Supplementary material for: Cloning and Characterization of the Gene Encoding HMGS Synthase in Polygonatum sibiricum
Source: Biomed Res Int. 2022 Oct 7;2022:7441296. doi: 10.1155/2022/7441296 (PMC9568320; doi:10.1155/2022/7441296)
Supplement: Supplementary Materials — Supplement: the sequences of the HMGS1 and HMGS2. [file 7441296.f1.docx]

**HMGS1 1470bp**

**>**

CGTTAATCGAAGGAGGAGAGATGATGGAGACGAGAGCTAAGGATGTTGGGATTCTTGCCATGGACATCTACTTCCCTCCCACTTGCGTTCAGCAGGAAGAGCTTGAAGCTCATGATGGGGCAAGCAAGGGGAAGTACACAATTGGACTGGGACAAGATTGCTTGGCCTTTTGCACAGAGGTGGAAGATGTCATCTCAATGAGCTTGACAGTTGTGACAACACTCCTTGAGAAGTATAAGATTGACCCTAAACAAATTGGGCGATTGGAAGTGGGCTCAGAGACGGTCATAGACAAGAGCAAGTCAATAAAAACTTGGCTCATGCAAATATTCGAGGAAAGTGGCAATACCGATATTGAAGGAGTTGATTCAACAAATGCATGCTATGGGGGAACAGCTGCTCTGCTCAACTGTGTAAATTGGGTGGAGAGTAACTCGTGGGATGGGCGTTATGGACTTGTTGTTTGTACAGACAGTGCGGTTTATGCAGAGGGCCCTGCTCGGCCAACCGGTGGTGCAGCTGCTATAGCAATGTTGATTGGGCCAAATGCTCCAATTGCTTTCGAGAGCAAGTATAGAGGGACTCACATGTCTCATGTTTATGATTTTTACAAGCCCAATCTAGCAAGTGAATATCCGGTTGTTGATGGAAAATTGTCACAAACGTGCTATCTCATGGCACTTGATTCATGCTACAAGCGGTTTTGTAACAAGTATGAGAAATTTGAGGGAAAACAGTTTTCAATTTCTGATGCAAATTATTTTGTATTTCACTCTCCATACAACAAGCTAGTGCAGAAAAGTTTTGCTCGGTTGTACTTCAATGACTTCTTGCGCAACTGCAGCTCTGTTGAGAAGGGAGCAAGAGAAAAGCTAGAGCCATTTTCATCCTTGTCTGGTGATGAAAGCTACCAAAGTCGCGACCTTGAAAAGGCTTCTCAACAAGTTGCCAAGAACTTGTACGATGAGAAGGTTCAACCATCCACTTTGGTACCAAAACAAGTGGGAAACATGTATACGGCATCCCTTTATGCAGCATTTGCATCTGTTCTTCACAACAAACATAGCACTCTGGTCGGTCAGCGGATCGTAATGTTCTCATATGGTAGTGGCTTGTCTTCCACGATGTTCTCATTCAGGATCCAGGATGGTCAGTATCCCTTCAGCATATCAAATATTGCTAATGTGATGAATGTCAATGGGAAGCTGGAGGCTAGGCATGTGTTTCCACCAGAAAAATTCGTTGAAACGATGAAGCTGATGGAGCACCGATACGGAGCCAAGGATTTCGTCACATCGAAAGACACAAGCCTGCTACCCCCTGGAACTTTCTATCTCACTGAGGTCGACTCCATGTACCGAAGGTTCTACACGAAGAAGGGCTCAGAAAGCAAATTGAGCGCTGCTGCTAACGGCATCATGGCCAATGGCCACTGATAATACTGCACGTATGCCAGTGGCATGGGTTTTG

**HMGS2 1416bp**

**>**

ATGATGGAGACGAGAGCTAAGGATGTCGGGATTCTCGCCATGGACATCTACTTCCCTCCCACTTGCGTTCAGCAGGAAGATCTAGAAGCTCACGATGGGGCAAGCAAGGGGAAGTATACAATTGGGCTAGGGCAAGATTGTATGGCCTTTTGCACAGAAGTGGAAGATGTCATCTCAATGAGCTTGACAGTTGTAACAACACTCCTTGAGAAGTTTAAGATTGATCCGAAACAGATTGGACGATTGGAAGTTGGTTCGGAGACGGTCATAGACAAGAGCAAGTCAATAAAAACTTGGCTTATGCAGATATTCGAGGATAGTGGTAATACTGATATTGAAGGAGTTGATTCAACTAATGCATGCTATGGGGGAACAGCTGCTCTGCTCAACTGTGTAAATTGGGTGGAGAGTAACTCGTGGGATGGGCGTTATGGACTTGTTGTTTGTACAGATAGCGCGGTTTATGCGGAGGGCCCAGCTCGGCCAACCGGCGGTGCAGCTTCTATAGCAATGCTGATTGGGCCAAATGCTCCAATTACTTTTGAGAGCAAATATAGAGGAACTCGCATGTCTCATGTTTATGATTTTTACAAGCCCAATCTAGCAAGCGAATATCCGGTTGTTGATGGAAAATTGTCACAAACATGCTATCTCATGGCACTTGATTCATGCTACAAGCGGTTTTGTAGCAAGTACGAGAAATTTGAGGGGAAACAGTTTTCAATTTCTGATGCAGATTATTTTGTATTCCATTCTCCATACAACAAGCTTGTGCAGAAAAGTTTTGCTCGGTTGTTCTTCAATGACTTCTTGCGCAACTGCAGCTCTGTTGAGAAGGGAGCAAGAGAAATGCTAGAGCCATTTGCATCCGTGTCTGGTGATGAAAGCTACCATAGTCGCGACCTTGAAAAGGCTTCTCAACATGCTGCCAAGAATTTGTACAATGAGAAGGTTCAACCATCTACTTTGGTACCAAAACAAGTGGGAAACATGTATACGGCATCCCTTTATGCAGCATTTGCATCTGTTCTTCACAACAAACATAGCACTCTGGTGGGTCAGCGAATCGTGATGTTCTCATATGGTAGTGGCTTGTCCTCCACAATGCTCTCATTCAAGATCCAGGATGGTCAGCATCCCTTCAGTATATCAAATATTGCTAGTGTGATGAATGTCAATGGGAAGCTGGAGGCTAGACATGTGTTTTCCCCAGGAAAATTTGTTGAAACGATGAAGCTGATGGAGCACAGATATGGAGCCAAAGATTTTGTCACATCGAAAGACACAAGCCTACTACCCCCTGGAACTTTCTACCTCACCGAGGTTGATTCCATGTACAGAAGGTTCTACGCGAAGAAGGGCTCAGAAAGCAAATCGAACGCTGCTGCGAACGGCACAATGGCCAACGGTCACTGA
